# Invertebrate behavior—actions or responses?

**DOI:** 10.3389/fnins.2013.00221

**Published:** 2013-11-19

**Authors:** Björn Brembs

**Affiliations:** Zoology - Neurogenetics, Universität RegensburgRegensburg, Germany

**Keywords:** decision making, animal, insects, mollusca, cricket, Drosophila, Aplysia, crustacean

Invertebrates have always been the reductionist neuroscientist's favorite. After all, are their nervous systems not simpler, their behavior not more stereotyped and reproducible than those of vertebrates, unfettered by cognition, and intelligence which would only serve to complicate the already tricky study of how neurons do the things they do? Until not too long ago, neurobiological study of invertebrate behavior seemed, by and large, to corroborate this view. We now believe we understand the giant fibers giving rise to fast escape behaviors from crustaceans, mollusks, or insects. We have discovered the central pattern generators controlling swimming in leeches, flying in locusts, feeding in mollusks, digesting in crustaceans, and walking in stick insects. We can now identify and characterize many of the neurons that process the visual stimuli prompting flies to turn, the courtship sounds attracting crickets and grasshoppers, or the olfactory stimuli enticing bees to extend their proboscis.

However, the apparent relative (to vertebrates) simplicity started to disappear, once scientists began to either omit parameters from the traditional experiments, add additional ones, or simply look more closely. This research topic highlights a selection of experiments which serve to demonstrate the kind of decision-making that is taking place even in invertebrates as soon as the experiment allows for sufficient degrees of freedom.

A mind-blowing list of recent examples of the kind of revelations scientists discover when they start to look in greater detail at phenomena we thought we understood is provided in Herberholz and Marquart (2012). Starting from the well-known giant fiber escape circuits in crustaceans, mollusks and insects, they show that today the role of these giant fiber systems is either questionable or only a small part of a range of different escape maneuvers with a large variety of different neural systems subserving them.

The uncrowned champions of reducing simple systems even more by eliminating as many confounding factors as possible must be the mollusks. In their riveting, neuron-by-neuron account of how even isolated ganglia of the marine snail *Aplysia* make spontaneous decisions and incorporate environmental feedback in this process of adaptive behavioral choice, Nargeot and Simmers (2012) elucidate principles of operant conditioning, habit formation, and compulsivity at a level of biological detail that will take decades to reach in most other systems.

Experiments in which parameters have been added to the traditional stimulus and its response can be grouped into two classes, those in which the internal state of the animal is taken into account and those in which stimuli are provided such as to establish a choice situation. The former class includes experiments described by Heinrich et al. (2012) on the neuronal and hormonal mechanisms influencing the decision to sing in different stages but under otherwise identical external circumstances in grasshoppers. Gaudry and Kristan (2012) explain in impressive detail the mechanisms by which different states of the medicinal leech exert their top-down influence on the processing of sensory stimuli at different stages of sensory processing, depending on the state of the animal. Far from simply being relayed to “higher” centers of the nervous system, from the sensory neurons onwards, other information is constantly being cross-correlated with and related to the sensory stream. While the coding properties of sensory neurons are the focus of Marsat and Pollack's (2012) review on ultrasound avoidance in crickets—neuronal bursts encode a “danger signal” from ultrasound often emitted by hunting bats—the work they review also shows that the final decision to initiate evasion behavior in crickets is formed in the brain of the animals, two to four synapses downstream of the sensory neurons that encode the “danger signal.” Analogously, Hirayama et al.'s (2012) contribution on the predatory sea-slug *Pleurobranchea* details the neural processes by which the animal's satiation state regulates approach/avoidance behavior.

The simplest way to add a second parameter to a traditional stimulus-response experiment is to choose a stimulus that allows two different responses, even if the state of the animal is not altered. Ritzmann et al. (2012) describe such experiments in which cockroaches must decide on which side (left vs. right or above vs. below) of an obstacle to proceed. The behavior of the animals is best being described as a value-based decision in which the needs of the animal (e.g., shelter) are negotiated with the ease of mastering the barrier. This value-based negotiation of rivaling incentives for an animal was also described in Herberholz and Marquart's (2012) account of crayfish negotiating simultaneous appetitive (food) and aversive (predator) stimuli of different relative value. This well-known cost/benefit tradeoff often encountered by animals in non-laboratory circumstances was also explicitly modeled in Hirayama et al. (2012). A further step away from the simpler, traditional experiments is to not only provide choices between stimuli or behaviors, but to integrate these with variations in the state of the animal. Itskov and Ribeiro (2013) describe experiments with fruit flies deciding about whether, what and when to eat. Due to rigorous behavioral experiments combined with *Drosophila*'*s* genetic tool arsenal, the neuronal and molecular mechanisms underlying the processes with which various external stimuli interact with different satiation states are slowly unraveling. Probably the most complex, most difficult to control and hence most challenging class of experiments are those where the outcome of the experiment determines the state of the animal and the stimuli are attached to other animals. Stevenson and Rillich's (2012) review of their work in cricket aggression begins to elucidate some of the neuronal components involved in mediating the simultaneous influence of experience, motivation, and sensory stimuli on the decision to fight or flee.

Perhaps to some the least surprising, but nevertheless most impressive decision-making performance can be reported from hymenopterans, arguably one of the smartest classes of invertebrates, perhaps only with a close rival in cephalopods (which are sadly not represented in this research topic). Wolf et al. (2012) remind us that the well-known navigational capabilities of desert ants are only a small aspect of their sophisticated and flexible food search and retrieval strategies. Of course, a research topic on invertebrate decision-making would not be complete without everybody's poster child for arthropod intelligence, the honey bee. In a tour de force, Zhang et al. (2012) lead us through a maze of different experiments showcasing the many levels of abstraction these animals can deploy in order to make adaptive foraging decisions. Probably among the conceptually deepest contributions is Jeanson et al.'s (2012) overview on collective decisions. Analogous to the super-organism concept of eusocial insects, it is tempting to transfer the factors guiding the emergence of a collective decision of individual invertebrates (e.g., noise amplified by positive feedback) and test if neurons in a decision-making circuit in a brain follow analogous rules when generating decisions such as the ones described above. Bringing us back to reductionism, as documented by Jeanson et al. (2012), these factors were identified mainly by reducing the contribution of the environment and relying only on the decision-making capabilities inherent in the individual animals themselves.

## References

[B1] Gaudry Q. Kristan W. B. (2012). Decision points: the factors influencing the decision to feed in the medicinal leech. Front. Neurosci. 6:101 10.3389/fnins.2012.00101 22783162 PMC3390556

[B2] Heinrich R. Kunst M. Wirmer A. (2012). Reproduction-related sound production of grasshoppers regulated by internal state and actual sensory environment. Front. Neurosci. 6:89 10.3389/fnins.2012.00089 22737107 PMC3381836

[B3] Herberholz J. Marquart G. D. (2012). Decision making and behavioral choice during predator avoidance. Front. Neurosci. 6:125 10.3389/fnins.2012.00125 22973187 PMC3428584

[B4] Hirayama K. Catanho M. Brown J. W. Gillette R. (2012). A core circuit module for cost/benefit decision. Front. Neurosci. 6:123 10.3389/fnins.2012.00123 22969700 PMC3431595

[B5] Itskov P. M. Ribeiro C. (2013). The dilemmas of the gourmet fly: the molecular and neuronal mechanisms of feeding and nutrient decision making in Drosophila. Front. Neurosci. 7:12 10.3389/fnins.2013.00012 23407678 PMC3569668

[B6] Jeanson R. Dussutour A. Fourcassié V. (2012). Key factors for the emergence of collective decision in invertebrates. Front. Neurosci. 6:121 10.3389/fnins.2012.00121 22933990 PMC3422758

[B7] Marsat G. Pollack G. S. (2012). Bursting neurons and ultrasound avoidance in crickets. Front. Neurosci. 6:95 10.3389/fnins.2012.00095 22783158 PMC3387578

[B8] Nargeot R. Simmers J. (2012). Functional organization and adaptability of a decision-making network in aplysia. Front. Neurosci. 6:113 10.3389/fnins.2012.00113 22855670 PMC3405415

[B9] Ritzmann R. E. Harley C. M. Daltorio K. A. Tietz B. R. Pollack A. J. Bender J. A. (2012). Deciding which way to go: how do insects alter movements to negotiate barriers? Front. Neurosci. 6:97 10.3389/fnins.2012.00097 22783160 PMC3390555

[B10] Stevenson P. A. Rillich J. (2012). The decision to fight or flee - insights into underlying mechanism in crickets. Front. Neurosci. 6:118 10.3389/fnins.2012.00118 22936896 PMC3424502

[B11] Wolf H. Wittlinger M. Bolek S. (2012). Re-visiting of plentiful food sources and food search strategies in desert ants. Front. Neurosci. 6:102 10.3389/fnins.2012.00102 22783163 PMC3389614

[B12] Zhang S. Si A. Pahl M. (2012). Visually guided decision making in foraging honeybees. Front. Neurosci. 6:88 10.3389/fnins.2012.00088 22719721 PMC3376410

